# Skeletal muscle twitch relaxation kinetics following heat stress endurance: carbohydrate versus placebo

**DOI:** 10.1007/s00421-026-06368-6

**Published:** 2026-07-21

**Authors:** Camila Nassif, Jack Cannon, Frank E. Marino

**Affiliations:** 1 School of Allied Health, Exercise and Sports Sciences, Charles Sturt University, NSW Orange, 2800 Australia; 2 School of Rural Medicine, Charles Sturt University, Orange, NSW 2800 Australia

## Abstract

**Purpose:**

To compare post-exercise twitch contractile properties and M-wave characteristics after 60-km self-paced cycling in the heat following placebo (PLA) or 6% carbohydrate (CHO) ingestion as beverages or capsules.

**Methods:**

Ten well-trained male cyclists completed five randomised Latin-square trials: water (H₂O), CHO beverage (CHO_b_), PLA beverage (PLA_b_), CHO capsules (CHO_c_), and PLA capsules (PLA_c_). Neuromuscular function was assessed before and after the time trial, and after a sustained knee-extensor contraction at 20% MVC to task failure. Measures included MVC, voluntary activation (VA), M-wave amplitude, evoked twitch properties, and integrated electromyography (iEMG).

**Results:**

Performance time (*p* = 0.336) and time to task failure (*p* = 0.394) did not differ between conditions. iEMG increased during the 20% MVC in all trials (all *p* < 0.0001). There were no condition × time interactions for MVC, VA, M-wave amplitude (all *p* ≥ 0.335), or twitch contractile variables (all *p* ≥ 0.115). Time effects were observed for rate of relaxation, which decreased in all trials (*p* = 0.0302 to *p* = 0.000239), contraction duration, which increased in all trials (*p* = 0.0412 to *p* = 0.000091), and half-relaxation time, which increased in H₂O, PLA_c_ and CHO_c_ (*p* ≤ 0.00322).

**Conclusions:**

Prolonged self-paced cycling in the heat produced a reproducible neuromuscular response dominated by slowed twitch relaxation. However, 6% CHO ingestion did not clearly attenuate these changes compared with PLA, suggesting that the principal impairment reflected exercise and heat load rather than CHO availability or delivery mode.

## Introduction

Carbohydrate (CHO) ingestion is a common nutritional strategy used by endurance athletes to delay fatigue and support performance, particularly during prolonged exercise where exogenous CHO can help maintain CHO oxidation and prevent hypoglycaemia (Jeukendrup 2004; Currell and Jeukendrup 2008; Skein et al. 2012). However, the ergogenic value of CHO is not uniform across protocols and contexts, and null effects are reported even under ecologically relevant cycling time-trial conditions (Nassif et al. 2008, 2014). In our previously published findings from a double-blind trial, ingestion of 6% CHO versus placebo (PLA), delivered either as a beverage or capsules with water, did not improve 60-km self-paced cycling time-trial performance in warm ambient conditions (32 °C, 50% RH) (Nassif et al. 2014).

Alongside ingestion studies, evidence from CHO mouth-rinse paradigms indicates that oral carbohydrate sensing can influence exercise performance via non-metabolic pathways (Gant et al. 2010; James et al. 2017; Kamaruddin et al. 2019; Hartley et al. 2022). Collectively, these findings strengthen the rationale to pair performance outcomes with mechanistic neuromuscular assessments when interpreting CHO versus PLA comparisons.

Recent work has renewed attention on the post-exercise period, where CHO intake is recommended to accelerate glycogen restoration and support capacity for subsequent endurance exercise (Gonzalez and Wallis 2021; Naderi et al. 2025). However, whether carbohydrate ingestion meaningfully accelerates recovery of skeletal muscle contractile function is less clear; carbohydrate supplementation can enhance muscle glycogen resynthesis yet not necessarily improve recovery of prolonged force depression, a functionally relevant marker of post-exercise weakness (Cheng et al. 2020). The present companion analysis extends our previous performance findings by examining neuromuscular responses measured during double-blind CHO–PLA trials. Specifically, we assessed whether CHO ingestion altered skeletal muscle contractile properties, voluntary activation, or muscle excitability before and after the 60-km time trial in the heat (Nassif et al. 2014), and again after a sustained 20% MVC contraction.

Neuromuscular fatigue during endurance exercise reflects interacting central and peripheral processes and can be experimentally separated using stimulation-based approaches applied at different sites along the pathway from the central nervous system to muscle force production (Gandevia 2001; Saboisky et al. 2003; Carroll et al. 2017; Newell et al. 2021). As exercise duration increases, fatigue may become relatively more “central” in origin and expressed through altered recruitment strategies that form part of an overarching pacing response (Ross et al. 2007). Additionally, peripheral contractile impairment remains relevant to functional capacity and recovery, and can be captured by post-exercise indices including muscle evoked potential (M-wave) characteristics and evoked twitch responses (Thomas et al. 2015; Newell et al. 2021).

Contemporary cycling studies show that prolonged cycling reduces MVC and alters M-wave properties post-exercise, while peak evoked torque may remain unchanged, consistent with complex interactions between neural drive, membrane excitability and excitation – contraction – coupling. CHO may aid glycogen restoration, but its effect on restoring contractile function is less clear (Jeffers et al. 2015; Newell et al. 2021). Given the mixed effects of CHO on endurance performance and post-exercise neuromuscular function, the inclusion of a sustained submaximal contraction following a long endurance trial might provide an additional mechanistic probe. That is, since sustained contractions are well-established for examining fatigue development because they produce progressive increases in muscle activation and a reproducible time-to-task-failure responses (Hunter and Enoka 2003) by providing sensitivity to changes in neural drive and contractile function not fully captured by brief post-exercise assessments. To provide a more sensitive assessment of potential CHO treatment effects beyond time-trial performance outcomes alone, a post-exercise sustained 20% MVC contraction was employed as a standardised probe of residual neuromuscular fatigue and exercise tolerance under controlled conditions. Specifically, we compared twitch contractile properties and associated M-wave responses following PLA, 6% CHO beverage, and 6% CHO capsule ingestion. We hypothesised that CHO ingestion would attenuate the development of peripheral fatigue during prolonged cycling, thereby resulting in attenuated reductions in neuromuscular performance following the sustained 20% MVC task.

## Methods

### Participants

Prior to testing and data collection, participants attended an information session outlining the nature of the research and possible risks involved and signed a letter of consent. The research was approved by the Ethics in Human Research Committee of the University (Protocol # 2005064). Ten male athletes (nine mountain bikers and one triathlete) volunteered to participate in the investigation. All had an extensive history of regular cycle training for at least three years prior to the study and completed a training distance of 350–400 km per week for the three months prior to data collection. Their physical characteristics were age 26 ± 3 years, mass 64.5 ± 7.7 kg, height 170 ± 9 cm, sum of nine skinfolds 54.2 ± 13.1 mm, peak power output 323 ± 41 W, and maximal oxygen consumption (VO_2max_) 70.7 ± 8.8 mL/kg/min. Skinfold sites were biceps, triceps, chest, sub axilla, subscapular, suprailiac, abdomen, thigh and calf. Participants performed a progressive VO_2max_ test using a Flow Trainer (TACX^®^, T1684, Netherlands) with their own bicycle attached in a thermoneutral environment (22 ± 1^**o**^C) using a previously described protocol (Kay et al. 2001).

### Experimental design and time-trial protocol

**Fig. 1:**
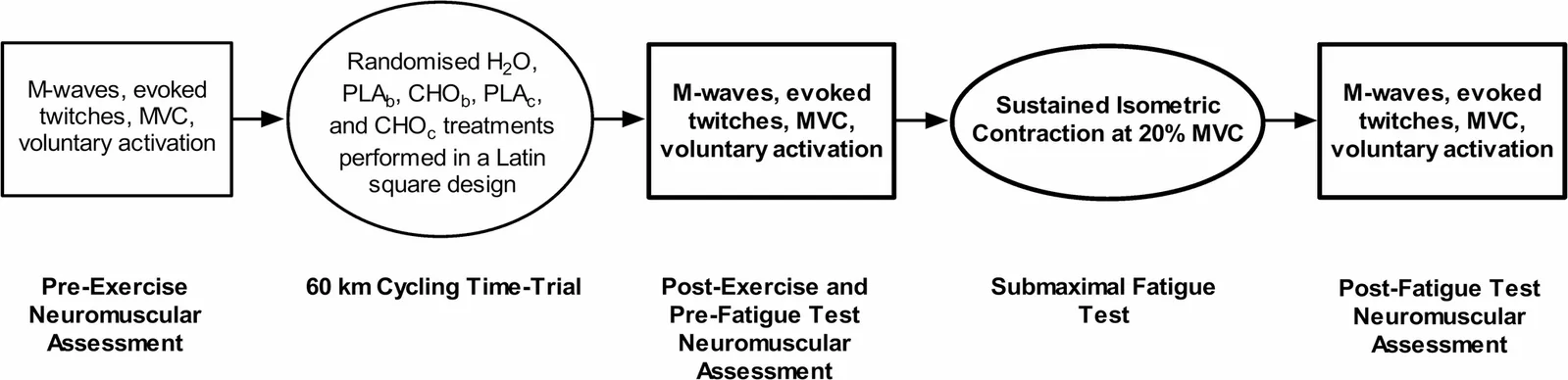
Outline of the experimental protocol and procedures used in the present study. The present study reports the effects of carbohydrate ingestion on parameters of neuromuscular fatigue following a sustained submaximal isometric contraction at 20% of the pre-exercise MVC following 60 km of self-paced cycling in the heat. For detals related to the 60 km self-paced trial see Nassif et al. (2014). MVC, maximal voluntary contraction, H_2_O; water treatment, PLA_b_; placebo beverage treatment, CHO_b_; carbohydrate beverage treatment, PLA_c_; placebo capsule treatment, and CHO_c_; carbohydrate capsule treatment

The experimental testing procedures used in the present study are graphically presented in Fig. 1. Using a randomized Latin square experimental design, participants completed a 60 km time-trial punctuated with 4 × 1 km sprints (at 14, 29, 44 and 59 km) on five separate occasions at the same time of day with each trial separated by 12 ± 7 days (Nassif et al. 2014). Before and after each time-trial, assessment for M-wave amplitude, evoked twitch contractile properties, maximal voluntary isometric torque (MVC), and the level of voluntary activation. Immediately following the post-exercise neuromuscular assessments, subjects performed a submaximal isometric fatigue protocol that required them to sustain 20% of their pre-exercise MVC for an extended period of time. Once the submaximal isometric protocol was terminated, the neuromuscular assessments were re-evaluated.

The methods related to the 60 km time trial have been described in detail elsewhere (Nassif et al. 2014). In brief, participants were instructed and encouraged to complete the 60 km distance as fast as possibly by completing the stage between sprints as quickly as possible and performing a maximal effort for the duration of each sprint. Trials took place in an environmental chamber (Russels^®^ Technical Products, model WMD-1150-5, Holland, MI, U.S.A.) in the heat (32 ^**o**^C and 50% relative humidity). Ambient temperature and relative humidity were controlled using the chamber’s integrated control system. During each trial, air movement through the chamber was provided by a fan positioned in front of the subject with a wind speed of 3 m/s in addition to the air circulation provided by the chamber.

For each of the trials, participants ingested either a placebo (PLA), 6% carbohydrate (CHO), or water (H_2_O) in a double-blind fashion. The PLA and CHO trials were each administered across two trials using different methods of ingestion. These were as a beverage (_b_) or in capsules (_c_) with distilled water. Therefore, each participant completed 2 x PLA trials (PLA_b_ and PLA_c_) and two CHO trials (CHO_b_ and CHO_c_). However, they were informed that ingestion was CHO for all beverage and capsule trials. The H_2_O trial was used as a control. The beverage and capsule conditions were included to separate potential oral-sensory effects from post-ingestive/metabolic effects of CHO. The beverage condition provided oral exposure to CHO, whereas the capsule condition delivered an equivalent CHO dose while minimising oral CHO sensing. PLA beverage and capsule conditions were included to preserve blinding and allow comparison across ingestion format. All trials other than H_2_O were coded and prepared by an individual not directly involved with the study.

Trial contents were revealed to the investigators at the completion of the study. Ingestion of fluids were scheduled at 5 km intervals, after each sprint (15, 30, 45 km) and at 55 km, and before the last sprint. Fluids were consumed at 4 ± 1 °C. Each participant ingested 284 ± 30 ml of beverage or 24 ± 3 capsules plus 284 ± 30 ml of distilled water at each of the intervals (4 ml per kg of body mass; (Hamilton et al. 1991). For each participant, the CHO powder in beverage and the number of capsules consumed with distilled water were carefully calculated to ensure the mixture corresponded to a 6% CHO solution. The CHO and PLA treatments were prepared by a commercial chemist (Stenlake^®^, Sydney, NSW, Australia) to ensure PLA_b_ and CHO_b_ matched in colour and flavour.

### Pre-testing requirements

For two days prior to each trial, the diet consumed was 60–70% CHO, 1.2–1.7 g of protein/kg of body mass, and 20–25% of fat, which was prescribed individually by a dietitian. The prescribed diet was to allow recovery of muscle glycogen stores following the last day of training and prior to each trial. Although not required to fast overnight prior to each trial, participants were required to consume the same meal the night prior to, and the morning of, each trial. Individual training routines were maintained up to 24 h prior to each trial, after which participants were instructed to abstain from strenuous activity, caffeine, and alcohol consumption. The 2-day dietary standardization period included the 24 h caffeine withdrawal.

### Physiological measures

Hydration status was assessed using urine specific gravity (USG; handheld refractometer, 503 Nippon Optical Works, Japan) before and immediately after exercise. Pre-exercise USG values were within the euhydrated range (USG ≤ 1.020). Post-exercise USG values increased, as expected following prolonged exercise in the heat, but remained below the threshold generally used to indicate dehydration (USG ≥ 1.03**)**. Accordingly, participants commenced trials euhydrated and did not reach a USG value consistent with dehydration at the end of exercise (Armstrong 2005). Core temperature (T_c_) was measured using the CoreTemp™ Telemetry System (HQInc. Wireless Sensing System and Design, Palmetto, Florida, U.S.A.). The telemetry pill was ingested the night before the trial with advice about timing according to the participants bowel motion.

### Neuromuscular testing apparatus

All neuromuscular assessments were performed on the dominant knee extensors under isometric conditions using a custom testing device (Master Equipment, Belo Horizonte, Brazil). Testing was performed while participants were seated upright with the hip flexed at 90^o^ (0^o^ being full extension) and the knee flexed at 65^o^ (0^o^ being full extension) and secured in the chair via waist and shoulder straps. During all tests, arms were crossed against the chest to ensure that additional forces did not contribute to performance. The lever arm axis of the testing device was aligned with the lateral epicondyle of the femur with the lower leg attached to the lever arm 1 cm above the lateral malleolus of the ankle. Voltage signals from a force cell with a 100 kg capacity (model 601, Tedea-Huntleigh, Don Mills, Canada) were fed to a host computer and were A/D converted at 16-bit resolution (DAQCard-6024E, National Instruments, Austin TX, U.S.A), low-pass filtered at 5 Hz, and sampled at a rate of 1000 Hz using data acquisition software (DASYLab version 9.0, Dasytec USA Inc, Bedford NH, USA). Force cell specifications indicate it had a linear response to loading (± 0.02%) represented by the equation; f(x) = ax + b. The voltage signal from the force cell while unloaded was determined and multiplied by − 1. This value was used in the equation above (variable b) to zero the signal. Subsequently, a series of known loads were placed on the force cell and the corresponding voltages determined. The ratio between the increase in load applied to the force cell and the increase in the voltage signal generated was determined (variable a) and was used to convert force cell signals (variable x) into units of measurement (i.e. newtons; variable f(x)). These procedures were performed using data acquisition software (DASYLab version 9.0, Dasytec USA Inc, Bedford NH, USA).

### Electromyography

Electrography (EMG) signals from the vastus medialis (VM) and rectus femoris (RF) were captured during maximal evoked testing (M-waves) and EMG signals from the VM, RF and vastus lateralis (VL) were captured during the submaximal isometric fatigue protocol (voluntary EMG). EMG signals were detected using single-differential pre-amplified EMG electrodes (bar configuration 1 mm x 10 mm; interelectrode distance 10 mm; bandwidth of 20–450 Hz) (model DE-2.1, Delsys, Boston, MA, USA) linked to an amplifier where signals where acquired at a gain of 100 V/V for M-waves or 1000 V/V for voluntary EMG with a common mode rejection ratio > 90dB (model Bagnoli-4, Delsys, Boston, MA, USA). Electrodes were positioned on the muscles according to SENIAM recommendations (Hermens et al. 2000). Skin was first prepared by shaving, abrading of the outer layer of epidermal cells, and removing of oil and dirt using an alcohol swab. For the vastus medialis, the recording electrode was attached at the distal 4/5 point along the line between the anterior superior iliac spine and the joint space in front of the anterior border of the medial ligament at the knee. For the rectus femoris, the recording electrode was attached halfway along the line between the anterior superior iliac spine and the superior border of the patella. For the vastus lateralis, the recording electrode was attached at the distal 2/3 point along the line between the anterior superior iliac spine to the lateral border of the patella. A disposable gel adhesive electrode was used as a reference (#2330, 3 M, Red Dot, St. Paul, MN, USA), which was positioned on the acromial process of the right shoulder. Signals were fed to a host computer (DAQCard-6024E, National Instruments, Austin TX, USA) and sampled at a rate of 2000 Hz for M-wave amplitude using EMGWorks Signal Acquisition software (version 3.0, Delsys, Boston, MA, USA.) and 1000 Hz for voluntary EMG during the submaximal fatigue protocol using DASYLab (version 9.0, Dasytec USA Inc, Bedford NH, USA).

For M-waves, muscle activation was achieved by percutaneous stimulation of the intramuscular branches of the femoral nerve using two 20 mm diameter disposable foam electrodes (Medi-trace 200 series, Kendall, USA) positioned about the medio-anterior aspect of the upper thigh directly below the inguinal fold and at the ventral portion of the vastus medialis. The current applied to the nerve was delivered by a constant-current stimulator (Digitimer DS7, Digitimer Ltd., Welwyn Garden City, Hertford-shire, UK) using a single square-wave pulse with a width of 200 µs (400 V with a current of 15–80 mA) linked to a host computer (DAQCard-6024E, National Instruments, Austin TX, USA). Initially, the current was manually applied in incremental steps until a muscle twitch of moderate amplitude was observed on the computer monitor. Stimulating electrodes positions were then adjusted medially and/or laterally until the site most responsive to stimulation was located. The electrodes were then secured in position and the location marked on the skin for future assessments. The stimulus intensity was gradually increased until a plateau in twitch amplitude was achieved, where the stimulus intensity was increased by 25% to ensure that supramaximal stimulation was delivered. For testing, five pulses, each separated by 10 s, were delivered with the subject at complete rest. This was determined based on the absence of EMG signals from the rectus femoris or vastus medialis and the absence of any load placed on the force cell other than that due to the effect of gravity on the lower leg. For processing, M-wave data for both RF and VM were averaged across all five trials with the mean analysed for peak-to-peak amplitude.

Voluntary EMG signals were sampled throughout the duration of the submaximal contraction. For processing, EMG signals were; (1) bandpass filtered with a low corner frequency of 20 Hz and a high corner frequency of 450 Hz using a second order Butterworth filter; (2) high-pass filtered at 15 Hz using a second order high-pass Butterworth filter to remove movement artefact (3) full wave rectified; then 3) low-pass filtered at 5 Hz to smooth the data. These procedures were performed using Labview software (version 8.0, National Instruments, Austin TX, USA). For analysis, EMG data were divided into six 5 s intervals relative to the time to fatigue. Interval 1 was obtained at the start of the contraction immediately subsequent to the attainment of the target torque (0% time to fatigue), interval 2 was obtained at 20% time to fatigue, interval 3 was obtained at 40% time to fatigue, interval 4 was obtained at 60% time to fatigue, interval 5 was obtained at 80% time to fatigue, and interval 6 was obtained during the final 5 s of contraction immediately prior to fatigue. Processed EMG data from each interval were integrated (iEMG) and normalised against the data obtained during interval 1. Thus, changes in iEMG over time were calculated as a perentage of interval 1using the equation; ∆iEMG (%) = (Interval 𝑥 / Interval 1) × 100, where interval 𝑥 is the interval examined (i.e. intervals 2–6). These procedures were performed using spreadsheet software for MS-Windows.

### Evoked twitch contractile properties

Immediately following M-wave testing, the dominate knee extensors were assessed by evoked twitch properties. Muscle activation was achieved by using the same electrode locations, testing apparatus, and stimulation parameters as previously described for assessing M-wave amplitude. For testing, five pulses, each separated by 10 s, were delivered with the subject at complete rest. This was determined based on the absence of EMG signals from the RF or VM and the absence of any load placed on the force cell other than that due to the effect of gravity on the lower leg. Force data were exported into spreadsheet software and multiplied by lever arm length to express data in units of torque (Nm). Torque data were then corrected for the effect of gravity on the lower leg, which was achieved by determining the average load applied to the force cell during the 50 ms period immediately before stimulation. Evoked torque-time curves were averaged across all trials with the mean used to determine; (1) peak twitch torque (Pt; defined as the highest isometric torque value achieved during the evoked contraction), (2) time to peak torque (TPt; defined as the time from torque onset to Pt), (3) half-relaxation time (½RT; defined as the time required for Pt to decline by half), (4) contraction duration (CD; TPt plus ½RT), (5) the rate of torque development (RTD; defined as the mean tangential slope of the twitch torque-time curve between the onset of torque development and Pt), and (6) the rate of relaxation (RR; defined as the mean tangential slope of the twitch torque-time curve between Pt and ½RT). For all evoked twitch contractions, torque onset was defined as the point at which torque data following stimulation increased beyond 2 SD of the mean torque calculated over the 50 ms period immediately before stimulation (Cannon et al. 2008).

### Maximal voluntary contraction

The assessment of maximal voluntary isometric torque (MVC) consisted of up to eight trials where subjects were instructed to attain peak force as fast as possible and continue exerting maximal effort for a period of 5 s. A minimum rest period of 30 s separated each trial and testing continued until the final three trials had values within ± 5% of each other. Strong verbal encouragement was provided during all voluntary efforts and subjects received continuous visual feedback of performance from a computer monitor. Force data were exported into spreadsheet software and corrected for the effect of gravity on the lower leg offline, which was achieved by determining the average load applied to the force cell during the 1 s period immediately before voluntary muscle contraction while the subject was at complete rest. The average load applied to the force cell during this period was used to offset the performance data obtained during testing. Following this, performance data were multiplied by lever arm length to express data in units of torque (Nm). MVC for each subject was determined as the single highest torque value produced among trials. A minimum of 30 s rest elapsed between trials. Strong verbal encouragement was provided during all voluntary efforts and subjects received continuous visual feedback from a computer monitor.

### Voluntary activation

Following the assessment of MVC, the level of voluntary activation of dominant knee extensors was assessed using the twitch superimposition technique (Saboisky et al. 2003). Muscle activation was achieved by using the same electrode locations, testing apparatus, and stimulation parameters used for assessing evoked twitch contractile properties. Testing consisted of a series of four trials whereby subjects received a superimposed stimulus during a MVC. For all trials, subjects were instructed to produce maximal effort as fast as possible and continue exerting maximal effort until instructed to relax, which was typically within 3–4 s. During each trial, the trigger for stimulation was manually primed ~ 1 s after initiation of each contraction. Once primed, the stimulus was automatically triggered when software detected a decline in peak force (DASYLab version 9.0, Dasytec USA Inc, Bedford NH, USA). Manually priming the trigger was necessary to prevent premature stimulation prior to the attainment of peak force. When primed, the decline in peak force necessary to automatically trigger the stimulus was < 1%. A minimum of 30 s rest elapsed between each trial. Strong verbal encouragement was provided during all voluntary efforts and subjects received continual visual feedback of performance from a computer monitor.

For analysis, force data were exported into spreadsheet software and corrected for the effect of gravity on the lower leg offline, which was achieved by determining the average load applied to the force cell during the 1 s period immediately before voluntary muscle contraction while the subject was at complete rest. The average load applied to the force cell during this period was used to offset the performance data obtained during testing. Following this, data were multiplied by lever arm length to express data in units of torque (Nm). Superimposed MVC was determined as the peak torque value produced during the 50–150 ms period after the delivery of the stimulus. Voluntary peak torque during each superimposed contraction prior to stimulation was determined as the mean torque value produced during the 25 ms before stimulus delivery. The level of voluntary activation using the following equation: Voluntary Activation (%) = (Voluntary peak torque/Superimposed torque) × 100. All trials were assessed with the trial yielding the highest level of voluntary activation used for subsequent analysis. It should be noted that due to the small decline (< 1%) in peak force necessary to automatically trigger the stimulus during the superimposed contractions, the level of voluntary activation may be underestimated. However, this decline is less than the variance observed in the measurement and is unlikely to have any meaningful impact on the data obtained.

### Submaximal fatigue test

**Fig. 2:**
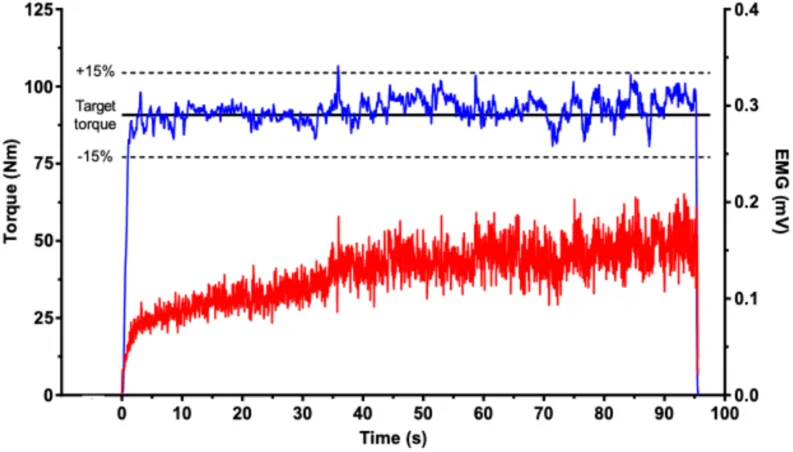
An example torque-time curve and EMG-time curve from the submaximal fatigue test from a representative participant. The target torque during the sustained submaximal contraction was 20% of the pre-exercise maximal isometric torque. The limits of maximum variation in torque output during the sustained contraction of ± 15% are identified

Immediately following the post-exercise neuromuscular assessments, subjects performed a submaximal fatigue test involving the dominant knee using the neuromuscular testing apparatus previously described. For this test subjects were required to sustain an isometric contraction at an intensity of 20% of their pre-exercise MVC until; (1) torque output was greater than ± 15% of the target torque required for a period of ≥ 3 s; or (2) the test was terminated by the subject due to voluntary fatigue. It should be noted that the first criterion was never met and all subjects terminated the testing due to voluntary fatigue. Subjects received strong verbal encouragement throughout all submaximal fatigue tests and were provided with continuous visual feedback of performance from a computer monitor. Performance was determined as the time to voluntary fatigue once the target torque value was attained, which was typically within 2–3 s after the initiating contraction. An example torque-time curve and EMG-time curve from a sustained submaximal contraction from a representative subject is provided in Fig. 2.

### Statistics analysis

Trials were completed using a randomised Latin-square crossover design so that order of conditions was counterbalanced across participants. This approach minimised systematic effects of trial order, familiarisation, training status, and residual fatigue across the five experimental conditions. As the present study involved secondary analyses that were not included in the original a priori power calculation, a sensitivity power analysis was performed in G*Power (v 3.9.1.6) for the repeated-measures neuromuscular design. Neuromuscular function was assessed at three time points: pre and post 60-km time trial and immediately following the sustained 20% MVC contraction. Each neuromuscular variable was treated as a separate dependent outcome. The mean observed correlation among repeated measures was *r* = 0.627. Using three repeated measurements, α = 0.05, desired power = 0.80, and *r* = 0.627, the analysis indicated that the study was sensitive to detect a moderate-to-large within-participant effect of *f* = 0.38. We, therefore, considered the analysis to have capacity to detect meaningful time-related neuromuscular changes.

Data were analysed using repeated-measures procedures appropriate to the structure of each dependent variable. Trial completion time and time to task failure during the sustained 20% MVC contraction were analysed using one-way repeated-measures ANOVA with condition as the within-participant factor: H₂O, PLA_b_, CHO_b_, PLA_c_ and CHO_c_. Neuromuscular variables measured across the experimental protocol were analysed using two-way repeated-measures ANOVA with condition and time as within-participant factors. Time points were pre and post-60 km, and post-sustained 20% MVC contraction. The iEMG response during the sustained contraction was analysed using repeated-measures ANOVA with condition and contraction interval as within-participant factors. Assumptions of sphericity were assessed using Mauchly’s test. Where violations were identified, Greenhouse–Geisser corrections were applied for statistical inference. Data distributions and model residuals were inspected for normality before inferential analyses. Where significant main or interaction effects were observed, post-hoc pairwise comparisons were performed using Fisher’s protected LSD. Effect sizes for ANOVA are reported as partial eta squared (η²), representing the proportion of variance in the dependent variable accounted for by model effects, with thresholds of 0.01 (small), 0.06 (moderate), and 0.14 (large). Pairwise effects are reported as mean differences with 95% confidence intervals and exact p values. Cohen’s dz was calculated from paired differences to provide a standardised practical measure of within-participant effect magnitude with values of 0.2, 0.5, and 0.8 representing small, moderate, and large effects, respectively. Statistical significance was accepted at *p* < 0.05. Statistical analyses were performed using IBM SPSS Statistics (v 31; IBM Corp., Armonk, NY, USA).

## Results

### Time trial performance and physiological measures

The results of the time trial performance and the associated physiological responses have been previously reported (see Nassif et al. 2014); however, here we include the results of the additional H_2_O trial, T_c_ and USG pre and post-exercise for context (Table 1). These data provide context for variability of performance and physiological responses across conditions. Pre-exercise USG values were within the range commonly considered euhydrated (USG ≤ 1.020). USG increased from pre- to post-exercise (*p* = 0.00026), as expected following prolonged exercise in the heat, but all post-exercise values remained below the threshold generally used to indicate dehydration (USG ≥ 1.030). Accordingly, participants commenced each trial euhydrated and did not reach a USG value consistent with dehydration at the end of exercise.

**Table 1:** The end exercise trial completion time and associated core temperatures and urine specific gravity

	H_2_O		PLA_b_		CHO_b_		PLA_c_		CHO_c_	
	Pre	End	Pre	End	Pre	End	Pre	End	Pre	End
Time (min)		138.2 ± 17.5		130.2 ± 11.2		140.5 ± 18.1		143.1 ± 29.2		137.3 ± 20.1
Range (min)		116.6–180.6		108.6-151.3		109.7–161.5		113.6–219.6		108.38–185.17
T_c_ (℃)	37.0 ± 0.2	38.0* ± 0.5	37.1 ± 0.2	38.1* ± 0.4	37.0 ± 0.3	38.2* ± 0.5	36.9 ± 0.4	38.0* ± 0.5	37.0 ± 0.2	38.2* ± 0.6
USG	1.0096 ± 0.0003	1.0156^#^ ± 0 0.0004	1.0104 ± 0 0.0005	1.0189^#^ ± 0.0005	1.0106 ± 0.0004	1.0189^#^ ± 0.0006	1.0118 ± 0 0.0004	1.0192^#^ ± 0.0007	1.0114 ± 0.0006	1.0170^#^ ± 0.0006

Pre is pre-exercise value and End is immediately post-exercise value. Time is minutes to complete the 60 km self-paced trial with completion time range referring to the minimum–maximum individual 60-km time within each condition, T_c_ is core temperature, USG is urine specific gravity and unitless. H_2_O is water, PLA is placebo and CHO is carbohydrate, b is beverage and c is capsule plus water. *p = 0.0001 and ^#^*p* = 0.00026 compared with pre-exercise, respectively. Values are means ± SD

### Submaximal fatigue test

**Fig. 3:**
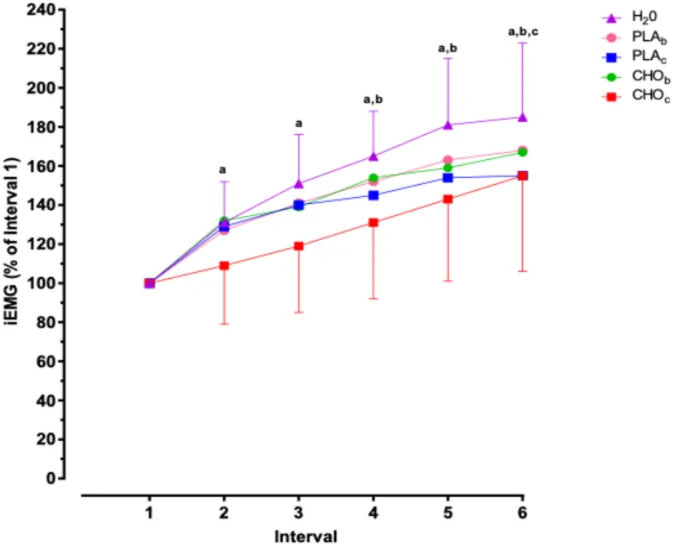
Change in iEMG amplitude during the sustained submaximal isometric contraction for each trial. Values are expressed as a percentage of interval 1 and presented as mean ± SD. Error bars have been removed from intermediate data points for clarity. ^a^Significantly different from interval 1 in the H_2_O, PLA_b_, PLA_c_ and CHO_c_ trials (all *p* ≤ 0.023). ^b^Significantly different from interval 1 in the CHO_b_ trial from interval 4 onward (*p* = 0.006). ^c^At interval 6, iEMG was significantly higher in H₂O than PLA_b_ (*p* = 0.042), CHO_b_ (*p* = 0.024) and CHO_c_ (*p* = 0.005). CHO, carbohydrate; PLA, placebo; b, beverage; c, capsule; H_2_O, water

Time to task failure for the sustained 20% MVC contraction did not differ between conditions (PLA_b_: 118.41 ± 101.65 s; PLA_c_: 103.09 ± 63.88 s; CHO_b_: 107.97 ± 72.28 s; H_2_O: 110.95 ± 76.80 s; CHO_c_: 101.01 ± 52.81 s; F(4,32) = 1.056, *p* = 0.394, partial η² = 0.117). The result was unchanged when Greenhouse–Geisser correction was applied (*p* = 0.361). The change in iEMG during the sustained submaximal contraction is presented in Fig. 3. There was a significant main effect of condition (F(4,36) = 4.488, *p* = 0.0048, partial η² = 0.333), a significant main effect of interval (F(5,45) = 77.605, *p* < 0.0001, partial η² = 0.896), and a significant condition × interval interaction (F(20,180) = 3.113, *p* < 0.0001, partial η² = 0.257).

Separate within-condition analyses showed significant increases in iEMG across the sustained contraction in all trials: H_2_O (F(5,45) = 52.332, *p* < 0.0001, partial η² = 0.853), PLA_b_ (F(5,45) = 24.080, *p* < 0.0001, partial η² = 0.728), CHO_b_ (F(5,45) = 33.230, *p* < 0.0001, partial η² = 0.787), PLA_c_ (F(5,45) = 35.876, *p* < 0.0001, partial η² = 0.799), and CHO_c_ (F(5,45) = 43.014, *p* < 0.0001, partial η² = 0.827). Post-hoc comparisons showed that iEMG was significantly greater than interval 1 from interval 2 onward in H₂O, PLA_b_, PLA_c_ and CHO_c_ (all *p* ≤ 0.023), whereas in CHO_b_ the increase reached significance from interval 4 onward (*p* = 0.0060). At the final interval, iEMG was higher in H₂O than PLA_b_ (mean difference 15.96%, 95% CI 0.69 to 31.23, *p* = 0.042, dz = 0.75), CHO_b_ (26.93%, 95% CI 4.51 to 49.35, *p* = 0.024, dz = 0.86), and CHO_c_ (24.11%, 95% CI 9.18 to 39.04, *p* = 0.005, dz = 1.16), but not PLA_c_.

The pre-trial MVC did not differ between conditions (F(4,36) = 1.719, *p* = 0.167, partial η² = 0.160). MVC, voluntary activation and M-wave amplitude across the three neuromuscular time points are presented in Table 2. There were no condition × time interactions for MVC (F(8,72) = 0.769, *p* = 0.631, partial η² = 0.079), voluntary activation (F(8,72) = 1.161, *p* = 0.335, partial η² = 0.114), RF M-wave amplitude (F(8,72) = 1.070, *p* = 0.394, partial η² = 0.106), or VM M-wave amplitude (F(8,72) = 0.914, *p* = 0.510, partial η² = 0.092). A main effect of time was observed for MVC (F(2,18) = 29.227, *p* = 0.000002, partial η² = 0.765), voluntary activation (F(2,18) = 3.569, *p* = 0.049, partial η² = 0.284), and RF M-wave amplitude (F(2,18) = 5.361, *p* = 0.015, partial η² = 0.373), but not VM M-wave amplitude (F(2,18) = 3.074, *p* = 0.071, partial η² = 0.255). Fisher’s LSD comparisons showed no significant change from post-60 km to post-submaximal contraction for MVC, voluntary activation, or RF/VM M-wave amplitude in any condition, with the smallest p value being *p* = 0.051 for VM M-wave amplitude in CHO_b_.

Evoked twitch contractile properties before and after the sustained submaximal contraction are presented in Table 3. There were significant main effects of time for Pt (F(2,18) = 22.496, *p* = 0.000013, partial η² = 0.714), RTD (F(2,18) = 33.901, *p* = 0.000001, partial η² = 0.790), RR (F(2,18) = 9.609, *p* = 0.00145, partial η² = 0.516), TPt (F(2,18) = 10.429, *p* = 0.000982, partial η² = 0.537), ½RT (F(2,18) = 30.277, *p* = 0.000002, partial η² = 0.771), and CD (F(2,18) = 21.962, *p* = 0.000015, partial η² = 0.709). No condition × time interactions were observed for any twitch variable (all *p* ≥ 0.115). Following the sustained contraction, Pt increased in PLA_b_ (*p* = 0.0397) and CHO_c_ (*p* = 0.0155). RR decreased in all trials: H₂O (*p* = 0.000239), PLA_b_ (*p* = 0.00950), CHO_b_ (*p* = 0.0302), PLA_c_ (*p* = 0.000539), and CHO_c_ (*p* = 0.00664). The ½RT increased in H₂O (*p* = 0.00322), PLA_c_ (*p* = 0.000210), and CHO_c_ (*p* = 0.000016), but not PLA_b_ (*p* = 0.0967) or CHO_b_ (*p* = 0.118). CD increased in all trials: H₂O (*p* = 0.00354), PLA_b_ (*p* = 0.00154), CHO_b_ (*p* = 0.0412), PLA_c_ (*p* = 0.000129), and CHO_c_ (*p* = 0.000091). Pairwise effect sizes for the significant twitch responses ranged in absolute magnitude from dz = 0.75 to 2.64, with the largest effects observed for prolonged ½RT in CHO_c_ and increased CD in PLA_c_ and CHO_c_.

**Table 2:** Maximal voluntary isometric torque, the level of voluntary activation, and M-wave amplitude Pre and post 60 km (pre-sub maximal contraction) and post submaximal fatigue test for each trial

	Trial	MVC (Nm)	VA (%)	M-wave Amplitude (mV)	
				RF	VM
Pre 60 km	H_2_O	178 ± 35	97.2 ± 3.3	5.8 ± 1.0	7.0 ± 0.9
	PLA_b_	169 ± 38	97.3 ± 2.3	5.2 ± 1.8	6.8 ± 1.7
	CHO_b_	172 ± 39	97.8 ± 1.4	6.5 ± 0.8	7.1 ± 0.8
	PLA_c_	180 ± 35	97.4 ± 2.1	5.8 ± 1.1	7.8 ± 0.1
	CHO_c_	168 ± 39	97.3 ± 3.8	6.4 ± 1.0	7.1 ± 0.9
	**Mean**	**171 ± 40**	**97.4 ± 2.6**	**6.0 ± 1.1**	**7.2 ± 0.9**
Post 60 km (Pre sub-max)	H_2_O	157 ± 42*	95.1 ± 5.2*	6.7 ± 1.0	7.3 ± 1.1
	PLA_b_	153 ± 38*	96.0 ± 3.8*	5.6 ± 1.7	6.6 ± 1.9
	CHO_b_	150 ± 43*	94.7 ± 8.2*	6.9 ± 0.4	7.9 ± 0.1
	PLA_c_	155 ± 36*	97.0 ± 3.1*	6.4 ± 0.7	7.5 ± 0.9
	CHO_c_	152 ± 40*	95.3 ± 7.6*	6.2 ± 0.9	7.2 ± 1.0
	**Mean**	**153 ± 40**	**95.6 ± 5.6**	**6.4 ± 1.0**	**7.3 ± 1.0**
Post sub-max	H_2_O	156 ± 77	96.1 ± 3.4	6.9 ± 1.0	7.4 ± 1.1
	PLA_b_	151 ± 42	95.7 ± 4.1	6.4 ± 1.2	7.6 ± 0.9
	CHO_b_	152 ± 39	96.5 ± 3.2	6.6 ± 0.7	7.5 ± 0.6
	PLA_c_	152 ± 31	96.8 ± 3.0	6.6 ± 0.7	7.7 ± 0.6
	CHO_c_	152 ± 39	94.6 ± 8.2	6.5 ± 0.9	7.5 ± 0.8
	**Mean**	**153 ± 36**	**96.0 ± 4.7**	**6.6 ± 0.9**	**7.6 ± 0.8**

MVC is maximal voluntary isometric torque, VA is the level of voluntary activation, RF is rectus femoris, VM is vastus medialis, H_2_O is water trial, PLA_b_ is placebo beverage, CHO_b_ is carbohydrate beverage, PLA_c_ is placebo capsule, CHO_c_ is carbohydrate capsule. Mean is across all trials. *p = 0.000002 compared with pre-exercise MVC and *p* = 0.049 compared with pre exercise VA; Values presented as mean ± SD

**Table 3:** Evoked twitch contractile properties before and after the submaximal fatigue test for each trial

	Trial	Pt (Nm)	RTD (Nm.s^− 1^)	RR (Nm.s^− 1^)	TPt (ms)	½RT (ms)	CD (ms)
Pre-submax	H_2_O	44.2 ± 10.7	708.1 ± 242.8	169.8 ± 36.9	72.1 ± 8.6	102.7 ± 14.5	174.8 ± 12.9
	PLA_b_	43.0 ± 11.5	742.7 ± 216.6	147.7 ± 41.9	70.9 ± 7.7	109.8 ± 14.8	174.5 ± 14.8
	PLA_c_	42.0 ± 10.6	737.0 ± 237.1	150.4 ± 37.7	70.4 ± 8.9	106.4 ± 12.3	170.9 ± 17.9
	CHO_b_	43.6 ± 10.4	704.1 ± 261.1	159.6 ± 53.3	75.0 ± 10.9	107.2 ± 23.7	182.2 ± 22.0
	CHO_c_	42.7 ± 10.2	764.5 ± 210.0	148.6 ± 31.8	68.4 ± 9.9	105.6 ± 11.3	167.2 ± 17.0
	**Mean**	**43.1 ± 10.3**	**731.3 ± 225.7**	**155.2 ± 40.2**	**71.4 ± 9.1**	**106.3 ± 15.4**	**173.9 ± 18.1**
Post-submax	H_2_O	44.5 ± 9.9	746.2 ± 238.9	130.5 ± 32.5*	75.9 ± 21.3	125.4 ± 25.1*	201.3 ± 25.2*
	PLA_b_	45.5 ± 11.4*	738.5 ± 260.3	122.1 ± 52.6*	79.9 ± 23.9	121.5 ± 13.9	201.4 ± 20.0*
	PLA_c_	43.5 ± 11.8	763.6 ± 223.9	126.3 ± 33.0*	69.5 ± 7.6	131.4 ± 16.3*	200.9 ± 15.7*
	CHO_b_	45.4 ± 11.0	675.0 ± 184.7	130.5 ± 36.4*	85.2 ± 25.0	123.9 ± 26.5	209.1 ± 20.1*
	CHO_c_	46.1 ± 10.9*	783.3 ± 242.3	128.7 ± 33.5*	73.1 ± 9.8	129.4 ± 15.5*	202.6 ± 22.0*
	**Mean**	**45.0 ± 10.6**	**741.3 ± 224.8**	127.6 ± 36.9*	**76.7 ± 19.0**	**125.1 ± 19.6**	203.1 ± 20.2*

Pt is peak twitch torque, RTD: rate of torque development, TPt: time to peak torque, RR: rate of relaxation, ½RT: half-relaxation time, CD: contraction duration, RF: rectus femoris, VM: vastus medialis, MVC: maximal voluntary contraction, VA: level of voluntary activation, H_2_O is water treatment, PLA_b_ is placebo beverage, CHO_b_ is carbohydrate beverage, PLA_c_ is placebo capsule, CHO_c_ is carbohydrate capsule, Mean; mean value across all treatment conditions. *Significantly different from Before within the same trial condition; *p* = 0.0412 to *p* = 0.000016. See text for individual exact p values. Values are presented as mean ± SD

## Discussion

The main finding of this companion analysis (Nassif et al. 2014) is that 60-km self-paced cycling in the heat was followed by a clear impairment in post-exercise twitch contractile properties, most consistently reflected by a slowing of relaxation. Yet these peripheral contractile responses were largely unchanged by 6% carbohydrate compared with placebo, whether CHO was delivered as a beverage allowing oral exposure or as capsules minimising oral CHO sensing.

Time to task failure during the sustained 20% MVC contraction did not differ between conditions, and there were no condition × time interactions for MVC, voluntary activation, M-wave amplitude, or any twitch contractile variable. Thus, although the protocol was sensitive to fatigue-related neuromuscular change, there was no clear evidence that carbohydrate altered the post-exercise neuromuscular profile. This distinction is a key finding where the study detected time-related effects, but these effects were not treatment-specific.

The most consistent contractile response was slowing of relaxation following the sustained contraction. Rate of relaxation decreased in all trials, CD increased in all trials, and 1/2RT increased in H₂O, PLA_c_ and CHO_c_. Slowed relaxation is a recognised feature of skeletal muscle fatigue and is commonly linked to changes in excitation–contraction coupling and Ca²⁺ handling (Allen et al. 2008). In contrast, MVC, VA and M-wave amplitude showed no systematic deterioration from post-60 km to post-submaximal contraction. This pattern suggests that the dominant measurable fatigue response in the present protocol was peripheral contractile slowing rather than impaired sarcolemmal excitability or reduced voluntary activation (Lepers et al. 2002; Carroll et al. 2017; Newell et al. 2021).

A notable result was the lack of a further decline in MVC after the sustained contraction. This contrasts with many rested-state submaximal task-failure protocols in which MVC often falls substantially at termination (e.g., ~ 42% after 20% MVC task failure), but it is plausible in the present design given (a) MVC was already depressed following the cycling bout and showed no evidence of recovery, and (b) fatigue response was perhaps captured more sensitively by twitch relaxation indices than by MVC. Post-exercise cycling studies that have used stimulation methods demonstrate that prolonged cycling can elicit detectable changes in evoked twitch properties and M-wave characteristics even when voluntary measures provide an incomplete picture of the peripheral component (Lepers et al. 2002). However, in the present design, the contraction was performed after prolonged self-paced cycling in the heat, so MVC was already reduced after the 60-km trial. It is, therefore, plausible that the additional sustained contraction produced a clearer effect on twitch relaxation kinetics than on maximal voluntary torque. The increase in peak twitch torque in PLA_b_ and CHO_c_ is also consistent with post-activation potentiation partly offsetting fatigue related contractile impairment, reinforcing the need to interpret peak twitch torque alongside relaxation variables rather than in isolation.

Although CHO ingestion can influence performance via metabolic provision, there is also substantial evidence that oral carbohydrate sensing can modify exercise responses via central mechanisms (Gant et al. 2010). CHO mouth-rinsing has been associated with improved endurance performance and is thought to act via oral receptor stimulation that increases central drive to locomotor muscles, with supporting evidence from brain imaging and corticospinal excitability studies (Chambers et al. 2009; Jeffers et al. 2015). Notably, mouth-rinse studies have reported attenuation of neuromuscular fatigue despite no improvement in time-trial performance, (Jeffers et al. 2015) and small reductions in fatigue-related strength loss following CHO mouth rinse compared with PLA (Jensen et al. 2015). The present data show that CHO ingestion (beverage or capsules) did not meaningfully alter post-exercise neuromuscular fatigue or the dominant twitch-based fatigue variable; relaxation slowing. There was also no consistent evidence that oral exposure by CHO beverage conferred any advantage over capsules. This suggests that if any “sensing” effects were present, they did not translate into an attenuation of the peripheral contractile impairment as measured by our post-exercise twitch protocol.

The H_2_O trial provides a practical reference condition in which no CHO was provided and no PLA manipulation was attempted. The T_c_ increased similarly from pre- to post-exercise across all trials (Table 1), and the post-exercise neuromuscular profile following the sustained submaximal task was broadly comparable. That is, MVC, VA and M-wave amplitude were unchanged (Table 2), while twitch indices showed a consistent fatigue response dominated by slower relaxation (Table 3). Although iEMG at task failure was higher in H₂O than PLA_b_, CHO_b_ and CHO_c_, this isolated difference was not accompanied by differences in time to task failure or twitch outcomes. Since the H_2_O trial was not blinded, expectancy effects cannot be excluded. Nevertheless, the close similarity of the neuromuscular responses across H_2_O and the blinded conditions supports the interpretation that the dominant post-exercise contractile impairment in our model reflects the exercise plus heat load itself rather than CHO provision per se.

We acknowledge several limitations with our model. First, although the repeated-measures design was sensitive to moderate-to-large time-related neuromuscular effects, the study was not an equivalence trial and cannot exclude smaller treatment-specific differences. Second, the self-paced time-trial design improves ecological validity but may introduce variability in pacing, duration and exercise intensity across trials. The Latin-square design counterbalanced trial order, but individual variation remains inherent to this type of performance model. Third, voluntary activation was assessed before and after the sustained contraction rather than continuously during the task. A protocol allowing repeated or continuous assessment of central drive during a self-paced or self-modulated contraction may provide additional insight in future studies.

## Conclusions

Prolonged self-paced cycling in the heat produced a consistent post-exercise slowing of twitch relaxation indicative of peripheral contractile impairment, but 6% CHO ingestion (beverage or capsules) did not attenuate these neuromuscular changes compared with placebo. The findings suggest that, under the present fed and self-paced conditions, the principal post-exercise neuromuscular impairment reflected the exercise and heat load rather than carbohydrate availability or mode of ingestion.

## Data Availability

All data generated in this experiment are available in the article.

## Abbreviations

CD: Contraction duration
CHO: Carbohydrate
iEMG: Integrated electromyography
M-wave: Maximal evoked potential
MVC: Maximum voluntary torque
PLA: Placebo
Pt: Peak twitch torque
RR: Relaxation rate
RTD: Rate of torque development
½RT: Half relaxation time
Tc: Core temperature
TPt: Time to peak torque
USG: Urine specific gravity
VA: Voluntary activation

## References

[CR1] Allen DG, Lamb GD, Westerblad H (2008) Skeletal muscle fatigue: cellular mechanisms. Physiol Rev 88(1):287–332 10.1152/physrev.00015.2007 18195089

[CR2] Armstrong LE (2005) Hydration assessment techniques. Nutr Rev 63(suppl 1):S40–S54 10.1111/j.1753-4887.2005.tb00153.x 16028571

[CR3] Cannon J, Kay D, Tarpenning KM, Marino FE (2008) Reproducibility and changes in twitch properties associated with age and resistance training in young and elderly women. Scand J Med Sci Sports 18(5):627–635 10.1111/j.1600-0838.2007.00709.x 18208423

[CR4] Carroll TJ, Taylor JL, Gandevia SC (2017) Recovery of central and peripheral neuromuscular fatigue after exercise. J Appl Physiol 122(5):1068–1076 10.1152/japplphysiol.00775.2016 27932676

[CR5] Chambers ES, Bridge MW, Jones DA (2009) Carbohydrate sensing in the human mouth: effects on exercise performance and brain activity. J Physiol 587(Pt 8):1779–1794 10.1113/jphysiol.2008.164285 PMC2683964 19237430

[CR6] Cheng AJ, Chaillou T, Kamandulis S, Subocius A, Westerblad H, Brazaitis M, Venckunas T (2020) Carbohydrates do not accelerate force recovery after glycogen-depleting followed by high-intensity exercise in humans. Scand J Med Sci Sports 30(6):998–1007 10.1111/sms.13655 32187403

[CR7] Currell K, Jeukendrup AE (2008) Superior endurance performance with ingestion of multiple transportable carbohydrates. Med Sci Sports Exerc 40(2):275–281 10.1249/mss.0b013e31815adf19 18202575

[CR8] Gandevia S (2001) Spinal and supraspinal factors in human muscle fatigue. Physiol Rev 81(4):1726–1789 10.1152/physrev.2001.81.4.1725 11581501

[CR9] Gant N, Stinear CM, Byblow WD (2010) Carbohydrate in the mouth immediately facilitates motor output. Brain Res 1350:151–158 10.1016/j.brainres.2010.04.004 20388497

[CR10] Gonzalez JT, Wallis GA (2021) Carb-conscious: the role of carbohydrate intake in recovery from exercise. Curr Opin Clin Nutr Metab Care 24(4):364–371 10.1097/MCO.0000000000000761 33973552

[CR11] Hamilton MT, GonzaleZ-Alonso J, Montain SJ, Coyle EF (1991) Fluid replacement and glucose infusion during exercise prevent cardiovascular drift. J Appl Physiol 71(3):871–877 10.1152/jappl.1991.71.3.871 1757323

[CR12] Hartley C, Carr A, Bowe SJ, Bredie WLP, Keast RSJ (2022) Maltodextrin-based carbohydrate oral rinsing and exercise performance: systematic review and meta-analysis. Sports Med 52(8):1833–1862 10.1007/s40279-022-01658-3 PMC9325805 35239154

[CR13] Hermens HJ, Freriks B, Disselhorst-Klug C, Rau G (2000) Development of recommendations for SEMG sensors and sensor placement procedures. J Electromyog Kinesiol 10(5):361–374 10.1016/s1050-6411(00)00027-4 11018445

[CR14] Hunter SK, Enoka RM (2003) Changes in muscle activation can prolong the endurance time of a submaximal isometric contraction in humans. J Appl Physiol 94(1):108–118 10.1152/japplphysiol.00635.2002 12391034

[CR15] James RM, Ritchie S, Rollo I, James LJ (2017) No dose response effect of carbohydrate mouth rinse on cycling time-trial performance. Int j Sports Nut Exerc Metab 27(1):25–31 10.1123/ijsnem.2016-0111 27616732

[CR16] Jeffers R, Shave R, Ross E, Stevenson EJ, Goodall S (2015) The effect of a carbohydrate mouth-rinse on neuromuscular fatigue following cycling exercise. Appl Physiol Nutr Metab 40(6):557–564 10.1139/apnm-2014-0393 25923580

[CR17] Jensen M, Stellingwerff T, Klimstra M (2015) Carbohydrate mouth rinse counters fatigue related strength reduction. Int J Sport Nutr Exerc Metab 25(3):252–261 10.1123/ijsnem.2014-0061 25203506

[CR18] Jeukendrup AE (2004) Carbohydrate intake during exercise and performance. Nut 20(7–8):669–677 10.1016/j.nut.2004.04.017 15212750

[CR19] Kamaruddin HK, Ooi CH, Mündel T, Aziz AR, Che Muhamed AM (2019) The ergogenic potency of carbohydrate mouth rinse on endurance running performance of dehydrated athletes. Eur J Appl Physiol 119(8):1711–1723 10.1007/s00421-019-04161-2 31098832

[CR20] Kay D, Marino FE, Cannon J, St Clair Gibson A, Lambert M, Noakes TD (2001) Evidence for neuromuscular fatigue during high-intensity cycling in warm, humid conditions. Eur J Appl Physiol 84:115–121 10.1007/s004210000340 11394239

[CR21] Lepers R, Maffiuletti NA, Rochette L, Brugniaux J, Millet GY (2002) Neuromuscular fatigue during a long-duration cycling exercise. J Appl Physiol 92(4):1487–1493 10.1152/japplphysiol.00880.2001 11896014

[CR22] Naderi A, Rothschild JA, Santos HO, Hamidvand A, Koozehchian MS, Ghazzagh A, Berjisian E, Podlogar T (2025) Nutritional strategies to improve post-exercise recovery and subsequent exercise performance: A narrative review. Sports Med 55(7):1559–1577 10.1007/s40279-025-02213-6 PMC12297025 40221559

[CR23] Nassif C, Ferreira AP, Gomes AR, Silva LM, Garcia ES, Marino FE (2008) Double blind carbohydrate ingestion does not improve exercise duration in warm humid conditions. J Sci Med Sport 11(1):72–79 10.1016/j.jsams.2007.08.015 18023253

[CR24] Nassif C, Gomes AR, Peixoto GH, Chagas MH, Soares DD, Silami-Garcia E, Drinkwater EJ, Cannon J, Marino FE (2014) The effect of double–blind carbohydrate ingestion during 60 km of self-paced exercise in warm ambient conditions. PLoS ONE 9(8):e104710 10.1371/journal.pone.0104710 PMC4128743 25110952

[CR25] Newell ML, Macgregor LJ, Galloway SDR, Hunter AM (2021) Prolonged cycling exercise alters neural control strategy, irrespective of carbohydrate dose ingested. Trans Sports Med 4(1):88–99

[CR26] Ross EZ, Middleton N, Shave R, George K, Nowicky A (2007) Corticomotor excitability contributes to neuromuscular fatigue following marathon running in man. Exp Physiol 92(2):417–426 10.1113/expphysiol.2006.035972 17099060

[CR27] Saboisky J, Marino FE, Kay D, Cannon J (2003) Exercise heat stress does not reduce central activation to non-exercised human skeletal muscle. Exp Physiol 88(6):783–790 10.1113/eph8802611 14603378

[CR28] Skein M, Duffield R, Kelly BT, Marino FE (2012) The effects of carbohydrate intake and muscle glycogen content on self-paced intermittent-sprint exercise despite no knowledge of carbohydrate manipulation. Eur J Appl Physiol 112(8):2859–2870 10.1007/s00421-011-2253-0 22138866

[CR29] Thomas K, Goodall S, Stone M, Howatson G, St Clair Gibson A, Ansley L (2015) Central and peripheral fatigue in male cyclists after 4-, 20-, and 40-km time trials. Med Sci Sports Exerc 47(3):537–546 10.1249/MSS.0000000000000448 25051388

